# Current models in bacterial hemicellulase-encoding gene regulation

**DOI:** 10.1007/s00253-023-12977-4

**Published:** 2024-01-04

**Authors:** Jessica K. Novak, Jeffrey G. Gardner

**Affiliations:** Department of Biological Sciences, University of Maryland – Baltimore County, Baltimore, MD USA

**Keywords:** Carbon catabolite repression, Carbohydrate active enzyme, Extracytoplasmic function, Hemicellulose, Hybrid two-component systems, Transcription factor

## Abstract

**Abstract:**

The discovery and characterization of bacterial carbohydrate-active enzymes is a fundamental component of biotechnology innovation, particularly for renewable fuels and chemicals; however, these studies have increasingly transitioned to exploring the complex regulation required for recalcitrant polysaccharide utilization. This pivot is largely due to the current need to engineer and optimize enzymes for maximal degradation in industrial or biomedical applications. Given the structural simplicity of a single cellulose polymer, and the relatively few enzyme classes required for complete bioconversion, the regulation of cellulases in bacteria has been thoroughly discussed in the literature. However, the diversity of hemicelluloses found in plant biomass and the multitude of carbohydrate-active enzymes required for their deconstruction has resulted in a less comprehensive understanding of bacterial hemicellulase-encoding gene regulation. Here we review the mechanisms of this process and common themes found in the transcriptomic response during plant biomass utilization. By comparing regulatory systems from both Gram-negative and Gram-positive bacteria, as well as drawing parallels to cellulase regulation, our goals are to highlight the shared and distinct features of bacterial hemicellulase-encoding gene regulation and provide a set of guiding questions to improve our understanding of bacterial lignocellulose utilization.

**Key points:**

*• Canonical regulatory mechanisms for bacterial hemicellulase-encoding gene expression include hybrid two-component systems (HTCS), extracytoplasmic function (ECF)-σ/anti-σ systems, and carbon catabolite repression (CCR).*

*• Current transcriptomic approaches are increasingly being used to identify hemicellulase-encoding gene regulatory patterns coupled with computational predictions for transcriptional regulators.*

*• Future work should emphasize genetic approaches to improve systems biology tools available for model bacterial systems and emerging microbes with biotechnology potential. Specifically, optimization of Gram-positive systems will require integration of degradative and fermentative capabilities, while optimization of Gram-negative systems will require bolstering the potency of lignocellulolytic capabilities.*

## Introduction

The decomposition of plant biomass plays a significant role in environmental and biotechnological settings (Zhang et al. 2020). As the largest source of renewable carbon on the planet, the deconstruction of its polysaccharide components is heavily studied (Von Freiesleben et al. 2018; Michalak et al. 2020; Mhatre et al. 2022). Plant cell wall polysaccharides are broadly classified as either cellulose or hemicellulose. Cellulose polymers are exclusively comprised of glucose with a single linkage type (Gardner and Blackwell 1974). Alternatively, hemicelluloses possess greater linkage and sugar varieties which can include xyloglucans, xylans, mannans, arabinans, and pectins (Hoch 2007). This diversity in linkage and sugar type contributes to the insolubility and recalcitrance of plant cell walls, making them difficult to degrade (Holland et al. 2020).

Environmental bacteria and fungi are the central decomposers of this material (Pascoal et al. 2021), and produce carbohydrate-active enzymes (CAZymes) for its deconstruction (Henrissat et al. 2022). Considerable biochemical and bioinformatic research has organized CAZymes into classes and families based on amino acid sequences and are documented in the CAZy database (Drula et al. 2022). This resource has facilitated efforts to predict and sort novel CAZymes for evolutionary phylogeny studies of lignocellulose degradation (Aspeborg et al. 2012; Wu et al. 2023), as well as enzyme engineering for industrial applications (Chettri and Verma 2023; Jayachandran et al. 2023).

As bacterial lignocellulose degradation systems become more fully described, work has branched out to several new areas to include the regulation of CAZyme-encoding genes. While cellulase systems in both Gram-negative and Gram-positive bacteria have been reviewed (Liu et al. 2021; Ziles Domingues et al. 2022), there have been much fewer for hemicellulase systems because of the large number of substrates and enzymes required, as well as the assertion that carbon catabolite repression (CCR) is the dominant modulator of gene expression (Stülke and Hillen 1999). Despite these challenges, recent hemicellulase-encoding gene regulation studies have characterized novel systems that were leveraged to engineer a single bacterium capable of fully degrading and fermenting lignocellulose (Mhatre et al. 2022; Singhania et al. 2022).

The goal of this review is to consolidate previously summarized work for a single phyla (Grondin et al. 2017; Lee et al. 2020) and provide commentary on the current direction of regulation-based studies for genes encoding hemicellulases like xyloglucanases, xylanases, mannanases, arabinanases, and pectinases in both Gram-negative and Gram-positive bacteria. Furthermore, this review discusses the breadth of knowledge regarding CAZyme-encoding gene regulatory systems to include the recent influx of transcriptomic and computational studies that predict regulons specific to hemicellulase-encoding genes. We conclude with a few open questions and offer suggestions on promising future directions for studying the regulation of hemicellulase-encoding genes that may be of environmental or industrial interest.

## Canonical regulatory mechanisms for bacterial hemicellulase-encoding gene expression

**Fig. 1:**
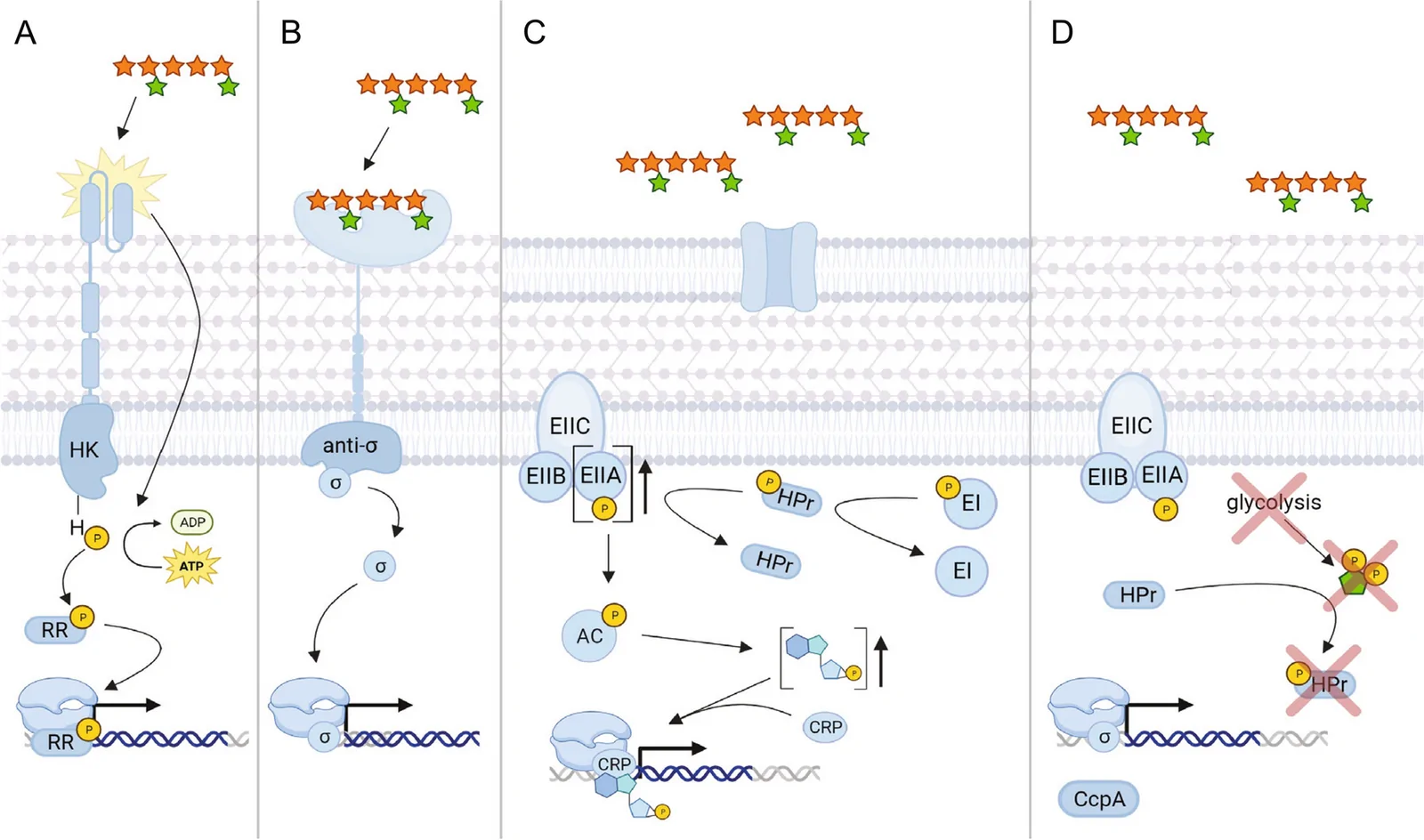
Common regulatory systems for Carbohydrate-Active Enzyme (CAZyme) encoding genes in Gram-positive and Gram-negative bacteria.** A** Hybrid two-component system in *Bacteroides thetaiotaomicron*. Upon sensing of arabinoxylan from the transmembrane domain, the intracellular histidine kinase (HK) phosphorylates the associated response regulator (RR) which recruits RNA polymerase for gene transcription. **B** ECF-σ/anti-σ factor system in *Bacteroides xylanisolvens*. Binding of arabinoxylan to the carbohydrate domain of the transmembrane ECF protein releases the intracellular σ factor from the membrane-attached anti-σ factor which aids RNA polymerase in gene transcription. **C** Carbon catabolite repression in Gram-negative *Escherichia coli*. In the absence of glucose, phosphorylated EII_A_ accumulates and activates adenylate cyclase (AC) via phosphorylation, which generates high cAMP levels. The cAMP subsequently binds to the cAMP receptor protein (CRP) and initiates transcription of hemicellulase-encoding genes. **D** Carbon catabolite repression in Gram-positive *Bacillus subtilis*. In the absence of glucose, fructose 1,6-biphosphate is not generated because glycolysis does not occur. Without fructose 1,6-biphosphate, histidine protein (HPr) does not get phosphorylated and therefore cannot dimerize with the carbon catabolite control protein (CcpA). Without this dimerization, the coupled protein cannot inhibit transcription. For all panels, phosphate is shown as a gold circle with a “P”; arabinoxylan is shown with orange stars for xylose and the green stars for arabinose” fructose is shown as a green pentagon. *Model generated with BioRender.com*

Expression of CAZyme-encoding genes requires precise regulation to ensure efficient energy expenditure under specific nutrient conditions. Despite the multitude of mechanisms that bacteria employ to regulate gene expression, there are only three systems commonly used for CAZyme-encoding genes, specifically hybrid two-component systems, extracytoplasmic function-σ/anti-σ systems, and carbon catabolite repression (Fig. 1). Given that these regulatory systems have been comprehensively reviewed previously (Liu et al. 2019; Pinto et al. 2019; Franzino et al. 2022), we will only briefly summarize each of their general functions and the current knowledge on these systems that is relevant for the expression of hemicellulase-encoding genes.

### Hybrid two-component systems

Hybrid two-component systems (HTCS) in bacteria use a sensing/phosphorylation relay mechanism to up-regulate genes involved in antibiotic resistance, virulence, biofilm formation, quorum sensing, and carbohydrate metabolism (Gutu et al. 2013; Cui et al. 2018; Gellatly et al. 2018; Kampik et al. 2020). This system, which is found in both Gram-negative and Gram-positive bacteria, recognizes an external stimulus with a cytoplasmic-membrane protein that initiates a phosphorylation cascade to modulate gene expression (Howell et al. 2003). As shown in Fig. 1A, a substrate binds the sensor domain of a transmembrane histidine kinase. Substrate binding initiates a phosphate transfer from ATP to a histidine residue on the cytoplasmic domain. The phosphorylated histidine kinase then transfers the phosphate to a response regulator which binds the transcriptional start site of interest to modulate transcription (Buschiazzo and Trajtenberg 2019; Francis and Porter 2019). It should be noted that there are examples of much lengthier phospho-relays with additional histidine kinases and response regulators before RNA polymerase recruitment. Two specific examples can be found in *Bacteroides thetaiotaomicron* and *Bacillus cereus* for glycan utilization and stress response, respectively (Been et al. 2006; Sonnenburg et al. 2006).

Previous research on hybrid two-component systems characterized the regulation of genes encoding xylanases, glucanases, arabinanases, and esterases from a diverse set of Gram-negative and Gram-positive bacteria (Emami et al. 2009; Martens et al. 2011; Shulami et al. 2014; Kampik et al. 2020). For example, in Gram-negative *Cellvibrio japonicus, Bacteroides thetaiotaomicron*, and Gram-positive *Ruminiclostridium cellulolyticum*, it was noted that HTCS regulators induced expression for biochemically or physiologically important xylanase-, arabinosidase-, and esterase-encoding genes (Emami et al. 2009; Martens et al. 2011; Kampik et al. 2020). The characterized HTCSs associated with xylanase and arabinanase-encoding gene expression are now cataloged as response regulators belonging to the AraC/XylS family of transcriptional activators (Emami et al. 2009; Celik et al. 2013). This family has recently been reviewed and is categorized based on two characteristic helix-turn-helix DNA-binding motifs (Cortes-Avalos et al. 2021). Regulation predominantly occurs via activation when the phosphorylated regulator binds to a recognized -10 and -35 consensus sequence upstream of the promoter for RNA polymerase recruitment (Celik et al. 2013). The sensing domains of these HTCS bind branched xylo-oligosaccharides or arabino-oligosaccharides in the periplasmic space for Gram-negative bacteria (Emami et al. 2009; Schwalm et al. 2017) and extracellularly for Gram-positive bacteria (Lansky et al. 2020). For the former, species like *C. japonicus* and *B. thetaiotaomicron* require an efficient mechanism to degrade extracellular hemicellulose into oligosaccharides and transport them to the periplasm where they can be sensed by the corresponding HTCS. It is therefore unsurprising that these two species possess a disproportionally high number of outer membrane transporters that can bring large complex oligosaccharides into the periplasm (Emami et al. 2009; Larsbrink et al. 2014; Blake et al. 2018; Pollet et al. 2021).

### Extracytoplasmic function (ECF)-σ/anti-σ systems

Similar to HTCS, extracytoplasmic function (ECF)-σ/anti-σ systems are also comprised of a membrane-spanning sensory protein with a cytoplasmic regulatory protein partner that controls gene expression, with specific roles in bacterial virulence, stress response, and carbohydrate catabolism (Sun et al. 2004; Alvarez-Martinez et al. 2007; Wang et al. 2019a). ECF-σ/anti-σ systems are found in both Gram-negative and Gram-positive bacteria, but have been most well-characterized in Actinobacteria and human gut symbionts belonging to the *Bacteroides* phylum (Martens et al. 2009; Bahari et al. 2011; Huang et al. 2015; Despres et al. 2016a; Wang et al. 2019a). The anti-σ element of the system is a protein in the cytoplasmic membrane that binds a cytoplasmic ECF-σ protein (Helmann 2002) (Fig. 1B). Release of the ECF-σ protein occurs upon substrate binding, which can be a glycan, metal, or chemical stressor like limonene (Pudio et al. 2015; Marcos-torres et al. 2016; Goutam et al. 2017). The freed σ-factor then binds to RNA polymerase, forming a holoenzyme, and initiates transcription after binding a recognized consensus mRNA sequence (Bae et al. 2015).

In the context of carbohydrate catabolism, ECF-σ/anti-σ systems are prominent regulators in human gut symbionts, especially for the expression of genes encoding *O*-glycan-degrading enzymes (Martens et al. 2008). ECF-σ/anti-σ systems in *Bacteroides* species also frequently regulate genes encoding TonB-dependent transporters (*e.g.,* SusC/D) (Martens et al. 2009). Furthermore, Gram-negative *Cytophaga hutchinsonii* and Gram-positive *Clostridium thermocellum* also have well-characterized ECF-σ/anti-σ systems that regulate cellulase-encoding gene expression (Nataf et al. 2010; Sand et al. 2015; Wang et al. 2019a). In *C. thermocellum*, cellulosomes are assembled using at least six ECF-σ/anti-σ systems that are specific for distinct cellulolytic regulons (Ortiz de Ora et al. 2018; Ichikawa et al. 2022).

In contrast to what is known about ECF-σ/anti-σ system to control cellulase-encoding genes, the regulatory involvement of ECF-σ/anti-σ systems for hemicellulase-encoding genes is less understood. Using the best-described examples from Actinobacteria, ECF-σ/anti-σ systems have been placed into families based on the regulons they control (Huang et al. 2015). For example, ECF families 52 and 53 have been computationally predicted to possess a C-terminal fusion domain comprised of the anti-sigma factor sequence coupled with a transmembrane portion of the protein (Marcos-Torres et al. 2022). More interestingly, some ECF52 and ECF53 proteins also have computationally predicted glycosyl hydrolase catalytic domains and carbohydrate-binding domains (Huang et al. 2015; Pinto et al. 2019); however, experimental validation has yet to be performed. In *C. thermocellum*, xylanase-encoding genes are regulated by alternative sigma factors σ^I6^ and σ^I7^ and the vegetative promoter σ^A^ (Sand et al. 2015; Ichikawa et al. 2022). It was demonstrated that the vegetative σ^A^ provided basal expression of xylanase-encoding genes, while σ^I6^ and σ^I7^ were employed for stronger expression in the presence of xylans (Bahari et al. 2011; Sand et al. 2015). Furthermore, the characterization of *C. thermocellum* ECF-σ/anti-σ systems aided in the prediction of homologous regulators in related species like *Psuedobacteroides cellulosolvens*, specifically for a pectin-degrading regulon (Ortiz de Ora et al. 2018)*.*

### Carbon catabolite repression

The final canonical system, carbon catabolite repression (CCR), is widely known for controlling the preferential utilization of specific carbon sources (typically glucose) over others (Ammar et al. 2018). In contrast to HTCS and ECF systems, which work similarly in Gram-negative and Gram-positive bacteria, the CCR mechanism in Gram-negative is markedly different compared to Gram-positive bacteria (Kundig et al. 1964; Deutscher and Saier 1983). In Gram-negative bacteria, a phosphotransferase system is utilized wherein glucose is imported intracellularly and simultaneously phosphorylated by a component of the transport protein (EII_A_). Expression of non-glucose metabolizing genes has very low basal expression and requires activation (Fig. 1C). A phosphorylated EI protein transfers a phosphate group to the HPr protein, which in turn phosphorylates EII_A_. In the absence of glucose, there is an abundance of phosphorylated EII_A_ (EII_A_ ~ P), which activates adenylate cyclase (AC) via phosphorylation (Magasanik 1961; Feucht and Saier 1980). The resulting accumulation of cAMP activates the cAMP receptor protein (CRP) and increases the transcription of genes that encode the proteins responsible for the metabolism of non-preferred carbon sources.

In Gram-positive bacteria, expression of genes important to the metabolism of non-glucose sugars requires inactivation of the repressor catabolite control protein (CcpA) (Fig. 1D). This occurs in the absence of glucose wherein fructose 1,6-bisphosphate (FBP) is not generated because glycolysis is not occurring. Without FBP, histidine protein (HPr) cannot be phosphorylated and dimerized with CcpA to repress the transcription of genes involved in metabolizing non-preferred carbon sources (Deutscher and Saier 1983). It should be noted that CcpA can also act as a transcriptional activator for quorum sensing (*trpA*), stress response (*cidAB*), and export of excess carbon (*ackA*) in *Streptococcus pneumoniae*,* Streptococcus mutans*, and *Bacillus subtilis* respectively (Henkin 1996; Kim et al. 2019a). Additionally, other counter-examples of CCR in *Pseudomonas* sp. found preferential utilization of succinate, citrate, or aromatic compounds over glucose (Liu 1952; Basu et al. 2006).

One example of CCR-based regulation for hemicellulase-encoding genes is found in *Bacillus subtilis* and uses both CcpA and the repressor GmuR (Sadaie et al. 2008). Mannanase-encoding genes in *B. subtilis* are in an operon that also contains genes encoding substrate-specific transporters and metabolic enzymes. In the presence of cellobiose or mannobiose (and in the absence of glucose), expression of the mannan utilization operon occurs due to a lack of fructose 1,6-bisphosphate. This results in limited amounts of phosphorylated HPr, which is necessary for CcpA binding to the promoter region. Consequently, the mannanase-encoding genes are de-repressed. Mannanase-encoding genes are further regulated by the repressor GmuR, which requires phosphorylation via GmuA, a component protein of the phosphotransferase system (PTS) and a structural homolog to EII_A_ (Sadaie et al. 2008). Briefly, glucomannan disaccharides are imported and phosphorylated via the PTS (comprised of transport proteins GmuABC). Inverse to the processes described for carbon catabolite repression, the presence of glucomannan oligosaccharides results in an abundance of unphosphorylated GmuA. Consequently, GmuR cannot be phosphorylated, which results in the transcription of mannanase-encoding genes.

Co-regulation of arabinanase and xylanase-encoding genes are found in Gram-negative and Gram-positive bacteria, with two characterized repressors being AraR and XylR (Laikova et al. 2001; Rodionov et al. 2001). Both belong to the LacI family of transcriptional regulators and work in conjunction with CCR (Book et al. 2016; Ohashi et al. 2021; Rodionov et al. 2021). Co-regulation of xylanase and arabinanase genes provides an efficient means of streamlining gene expression given the monosaccharide composition of lignocellulose, namely hexoses coming from cellulose and pentoses coming from hemicellulose (Jamander et al. 2014; Kim et al. 2015). Not surprisingly, CCR has been widely studied to characterize the regulation of lignocellulose-derived sugar metabolism in *Clostridium*,* Caldicellulosiruptor*,* Pseudomonas*, and *Escherichia* species (Gosset 2005; Vanfossen et al. 2009; Bruder et al. 2015; Liu et al. 2015).

### Current applications of canonical systems

The use of bacteria as lignocellulose bioprocessors has renewed interest in the three canonical regulatory mechanisms for biotechnologically relevant bacteria (Mearls et al. 2015; Taylor et al. 2018; Elmore et al. 2020; Ling et al. 2022). Using HTCS and ECF-σ/anti-σ systems, recent studies have focused on the regulation of polysaccharide utilization loci (PULs) containing hemicellulase-encoding genes, especially in *Bacteroides* sp. (Luis et al. 2018; Mackie and Cann 2018; Pereira et al. 2021; Beidler et al. 2023). Similarly, *C. thermocellum* is commonly used to study ECF-σ/anti-σ systems due to it possessing unique σ^I^ factors that can be studied heterologously in *B. subtilis* (Munoz-Gutierrez et al. 2016). Comparative studies of *C. thermocellum* σ^I^ factors were also important to the discovery that transcriptional initiation of cellulosomal genes relied on an auto-proteolysis system for ECF upon binding to the glycan of interest (Chen et al. 2023a). Likewise, dismantling CCR-related mechanisms in biotechnologically relevant bacteria (*e.g., E. coli, C. thermocellum,* and *P. putida*) found that co-utilization of xylose and glucose is more easily achieved with intracellular cellobiose hydrolysis (Xiong et al. 2018; Wang et al. 2019b; Cabulong et al. 2021). Intracellular cellobiose hydrolysis and phosphorylation bypassed some of the inhibitory effects caused by bacterial sensing/detection of extracellular glucose. Moreover, *Pseudomonas putida* KT2440 has undergone extensive engineering to co-metabolize glucose with cellobiose, galactose, xylose, and arabinose (Dvorak and de Lorenzo 2018; Peabody et al. 2019; Elmore et al. 2020).

## Transcriptomic approaches to identify hemicellulase-encoding gene regulatory patterns

The use of transcriptomic data to assess global changes in CAZyme-encoding gene regulation has rapidly become a standard approach to identify critical components of polysaccharide degradation (Gruninger et al. 2018; Lillington et al. 2020; Chen et al. 2023b). This method is particularly useful for non-model bacterial systems whose regulatory mechanisms are less characterized compared to *E. coli* or *B. subtilis.* While it should be noted that CAZyme-encoding gene expression was previously known to be regulated by growth rate and bacterial life cycle for *Bacteroides succinogenes* and *Clostridium thermocellum* (Russell 1987; Rydzak et al. 2012), more recent reports have uncovered unique differences in hemicellulase-encoding gene regulation for both Gram-positive and Gram-negative bacteria. Below is a summarization of the recent developments using transcriptomics to elucidate regulatory features in lignocellulose-degrading bacteria.

### Hemicellulase gene expression in Gram-positive species

**Fig. 2:**
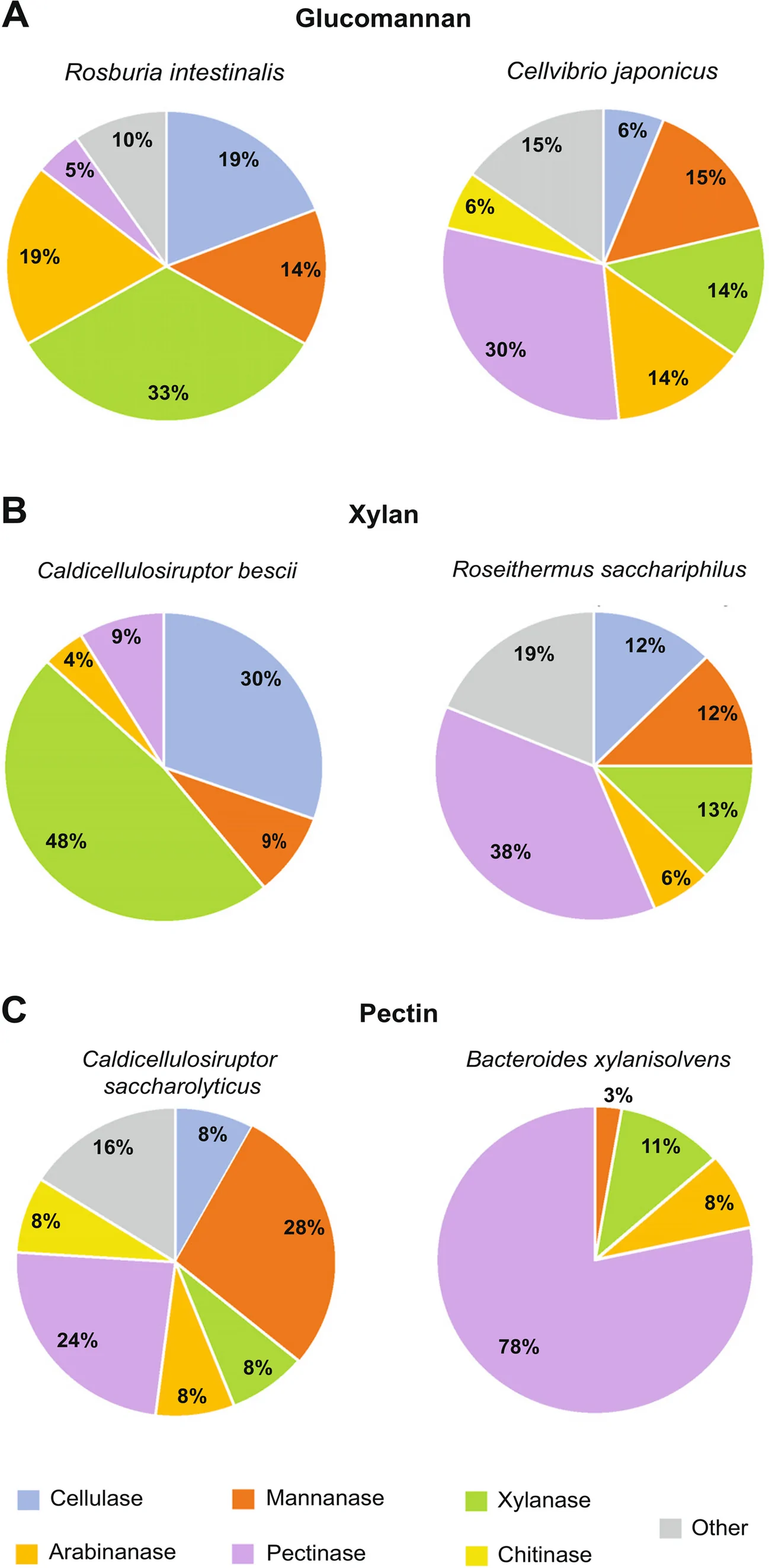
Differences in up-regulation of CAZyme-encoding genes from selected Gram-positive and Gram-negative bacteria when grown using hemicelluloses. **A** CAZyme-encoding gene expression of Gram-positive *Roseburia intestinalis* and Gram-negative *Cellvibrio japonicus* on glucomannan. **B** CAZyme-encoding gene expression response of Gram-positive *Caldicellulosiruptor bescii* and Gram-negative *Roseithermus sacchariphilus* on xylan. **C** CAZyme-encoding gene expression response of Gram-positive *Caldicellulosiruptor saccharolyticus* and Gram-negative *Bacteroides xylanisolvens* on pectin

Current RNAseq analyses using Gram-positive bacteria grown on hemicelluloses have often revealed highly specific gene expression responses (Blumer-schuette et al. 2017; La Rosa et al. 2019; Rodionov et al. 2021). For example, the human gut symbiont *Roseburia intestinalis* has a substrate-specific response during growth on glucomannan and galactomannan (Fig. 2A) (La Rosa et al. 2019). Notably, 16 up-regulated genes were from two distinct mannan utilization loci that differ from PULs described in *Bacteroides* by the absence of genes that encode TonB-dependent transporters. Additionally, *R. intestinalis* growth on galactose (a component of galactomannan) did not result in up-regulation of any of these genes, suggesting that mannose or manno-oligosaccharides were the sole nutritional signal for mannan deconstruction (La Rosa et al. 2019).

Highly specific CAZyme-encoding gene regulation has been observed in *Bacillus* sp. N16-5, where up-regulation of β-mannanase and α-galactosidase-encoding genes was only observed when the bacterium was grown using galactomannan, but not on xylan, pectin, CMC, or any tested monosaccharide (glucose, fructose, mannose, galactose, arabinose, or xylose) (Song et al. 2013). Furthermore, *Bacillus* sp. N16-5 grown using xylan only up-regulated β-xylanase-encoding genes, but growth on xylan or xylose up-regulated xylulokinase and xylose-related transporter-encoding genes.

As a third example, in *Caldicellulosirupter* species like *C. bescii* and *C. saccharolyticus*, xylanase-encoding genes were strongly up-regulated during growth on xylan (Fig. 2B) but repressed on either xylose or cellulose (Blumer-schuette et al. 2017; Rodionov et al. 2021). Expression data of *C. bescii* when grown using xylan also identified a putative key xylanase for extracellular xylan degradation (Xyn11A-2) (Crosby et al. 2022); however, a comparison of enzymatic activity between the *C. bescii* xylanases showed relatively mediocre activity for Xyn11A-2. The authors suspect this observed difference in gene expression could be a compensatory mechanism to overcome the modest activity of Xyn11A-2. The use of transcriptomic data from *C. bescii* when grown on xylan has also proven useful for pairing the important degradative loci to their likely regulat*o*rs, which included XynR, XylR, AraR, BxgRS, and AxuRS (Rodionov et al. 2021). Interestingly, transcriptomic analysis of *C. saccharolyticus* grown using pectin found a much broader gene expression response than that observed on other hemicelluloses (Blumer-schuette et al. 2017). Growth of *C. saccharolyticus* using pectin elicited up-regulation of various CAZyme-encoding genes, including cellulases, mannanases, xylanases, arabinanases, pectinases, and chitinases (Fig. 2C).

As a final example, *Clostridium* sp. exhibited some divergence in their regulatory circuits for xylanase-encoding genes (Petit et al. 2015; Munir et al. 2016). The expression of xylanase-encoding genes possessed by *C. termitidis* was dependent on xylan, but not xylose, cellobiose, or cellulose, while those belonging to *C. phytofermentans* were up-regulated when grown on both xylan and cellulose. Alongside the differences in hemicellulase gene expression observed between growth media, growth rate is also a critical mediator of CAZyme gene expression in *Clostridium* sp., with several studies reporting *C. thermocellum* transcription of cellulase-encoding genes dependent upon growth phase (Dror et al. 2003; Riederer et al. 2011). One interesting exception was for a xylanase-encoding gene (*xynC*), which exhibited high expression irrespective of growth rate (Dror et al. 2005).

### Hemicellulase gene expression in Gram-negative species

For Gram-negative bacterial species, transcriptomic studies have revealed much broader gene expression responses than those observed in Gram-positive bacteria (Blake et al. 2018; Chen et al. 2018; Novak and Gardner 2023). For example, in *Leeuwenhoekiella* sp*.* MAR_2009_132 and *Salegentibacter* sp*.* Hel_I_6, up-regulated α- and β-mannanase-encoding genes were identified when these bacteria were grown on both α- or β-mannan despite the selective activity of these CAZymes for each substrate (Chen et al. 2018). This suggested that these species regulate mannanase gene expression with less specificity, possibly at the level of the mannose monosaccharide given that these bacteria cannot differentiate between α- versus β-mannan.

A broad gene expression response was revealed in the saprophyte *Cellvibrio japonicus* when grown on glucomannan (Fig. 2A) (Novak and Gardner 2023). Eight of the ten predicted mannanase-encoding genes were up-regulated, as well as an additional 46 CAZyme-encoding genes. Strong up-regulation of non-substrate specific CAZyme-encoding genes in *C. japonicus* suggests that it is likely the presence of complex polysaccharides that induce gene expression. Additionally, a previous study of the *C. japonicus* transcriptomic response on cellobiose also resulted in broader up-regulation of cellulases and hemicellulases (Nelson et al. 2017). Interestingly, a much more specific response was elicited when *C. japonicus* was grown on oat-spelt xylan (Blake et al. 2018). This report concluded that *C. japonicus* only up-regulated xylanase genes during mid-exponential growth, though a comparison of the RNAseq from the stationary phase showed up-regulation of genes encoding xylanases, arabinanases, mannanases, and cellulases. In terms of the growth rate affecting CAZyme-encoding gene expression in *C. japonicus*, it was observed that expression was more prominent during active growth compared to the stationary phase (Blake et al. 2018; Novak and Gardner 2023).

*Roseithermus sacchariphilus* exhibited a transcriptomic response quite dissimilar to *C. japonicus* when it was grown on beechwood xylan (Liew et al. 2020). This bacterium had up-regulation of genes encoding cellulases, mannanases, xylanases, arabinanases, pectinases, and other glycosidases (Fig. 2B). Surprisingly, pectinase-encoding genes were the most prominently up-regulated CAZyme-encoding genes when *R. sacchariphilus* was grown on xylan. The authors hypothesize that the broad response was due to co-expression of genes encoding various glycosidic activities by the same promoter. However, they also suggested that a multi-timepoint transcriptomic analysis could reveal more about the patterns of hemicellulase gene expression.

Finally, expression of CAZyme-encoding genes in *Bacteroides xylanisolvens* also elicited a broad gene expression response on oat-spelt xylan, with up-regulation of 150 carbohydrate utilization-encoding genes that included all identified PULs for xylan utilization and 15 PULs for starch and pectic metabolism (Despres et al. 2016a). The authors hypothesized that the broad response was from the detection of shared oligosaccharides present in both oat-spelt xylan and pectins (*i.e.,* arabinoside side-chains). However, this response was very different when *B. xylanisolvens* was grown on citrus pectin and resulted in a much more specific result (Fig. 2C) (Despres et al. 2016b). Here, researchers were able to compare the gene expression response on two different types of pectins and discern the PULs that were most likely to be involved in the degradation of different pectic linkages. Specifically, PUL 2 was suspected to be important to degrading type II rhamnogalacturonan, PUL 13 was likely involved in de-branching arabinose sidechains, and PULs 49 and 50 were the most up-regulated on both pectins and were suspected to be involved in degrading homogalacturonan and type I rhamnogalacturonan, respectively. Additionally, *B. xylanisolvens* shared the traits observed in other bacterial species with high expression of CAZyme-encoding genes during active growth compared to the stationary phase (Despres et al. 2016b).

### Hemicellulase gene expression in bacterial co-culture

There has been increasing interest in the metatranscriptomic of co-cultured bacteria using complex polysaccharide-rich substrates given that environmental lignocellulose degradation is performed by a microbial community. For example, a study of the Gram-positive *Butyrivibrio hungatei* MB2003 transcriptome during mono- and co-culture with rumen gut symbiont *Butyrivibrio proteoclasticus* B316 found that in monoculture, *B. hungatei* was unable to grow on xylan or pectin despite the presence and expression of several hemicellulase-encoding genes (Palevich et al. 2019). Strikingly, when in co-culture with *B. proteoclasticus*, *B. hungatei* had a substantial increase in its growth capabilities at the expense of *B. proteoclasticus* final cell density. Since *B. hungatei* acts more as a sugar scavenger than a hemicellulose-degrader, its RNAseq results in monoculture unsurprisingly showed marked increases in the expression of many genes important to translation, signal transduction, defensive mechanisms, lipid/amino acid metabolism, and cell wall biogenesis compared to its co-cultured counterpart. During co-culture with *B. proteoclasticus*,* B. hungatei* expressed fewer genes overall but exhibited more specificity in the expression of genes encoding for carbohydrate metabolism (*e.g.,* ABC sugar transporters). Interestingly, *B. proteoclasticus* gene expression was relatively unchanged between mono- and co-culture (excluding a few CAZyme-encoding genes which were up-regulated during co-culture) despite the increase in competition provided by culturing with *B. hungatei*.

As another example, the Gram-negative gut symbionts *P. intestinalis*,* P. muris*, and *P. rodentium* underwent comparative metatranscriptomic analysis, and the study concluded that *P. intestinalis* was the most competitive strain due to its distinct up-regulation of PULs encoding xylanase and pectinase-encoding genes when the rat host was given a diet heavy in arabinoxylans (Galvez et al. 2020). The three most up-regulated glycoside hydrolase families in all three species were from GH43, GH2, and GH28. These families contain members able to hydrolyze β-glucan, β-xylan, α-arabinan, and pectic linkages (Lombard et al. 2014).

Co-cultures containing both Gram-positive and Gram-negative species have been used to investigate the bottlenecks of complete lignocellulose bioconversion in the guts of rumen or humans (Leth et al. 2018; Badhan et al. 2022). A recent metatranscriptomic study examined a complex consortium of Gram-positive and Gram-negative gut symbionts in ruminant animals grown in ex vivo batch culture on total tract indigestible residue (TTIR). The primary goal of the study was to assess the bottlenecks in ruminant digestion to uncover mechanisms to enhance the system. Transcripts encoding xylanases were abundant when the micro-community was grown on TTIR, which indicated that heteroxylans and xyloglucans were the primary remaining polysaccharide in the TTIR. It was hypothesized that the sheer quantity of inter- and intramolecular bonds act as a hindrance to enzyme accessibility to the substrate.

Overall, there appears to be a distinguishing difference between the hemicellulose-encoding gene expression patterns in Gram-positive versus Gram-negative bacteria. Specifically, the narrowed specificity of gene expression observed in Gram-positive compared to Gram-negative species. Additionally, investigations of co-culture transcriptomics containing Gram-positive and/or Gram-negative communities on lignocellulose have focused on the interspecies relationships and competition for carbon acquisition (Palevich et al. 2019; Galvez et al. 2020; Badhan et al. 2022). The knowledge obtained from these analyses has subsequently been applied in studies on gut microbiomes and biotechnology applications, specifically for studies that successfully predicted the impact of synthetic gut microbiota on host immune response (Afrizal et al. 2022) and identified patterns in microbe abundance based on diet (Corbin et al. 2023).

### Computational prediction of transcriptional regulators using compilations of transcriptomic data

In addition to the plethora of information provided by RNAseq data from a singular dataset, compilations of such data can extrapolate more information on transcriptional regulatory systems using computational methods. For example, transcriptomic compilations with DNA-binding motif studies have predicted extensive transcriptional regulatory networks of several different bacteria (Poudel et al. 2020; Rychel et al. 2020). The known computationally predicted regulons of Gram-negative plant bioprocessors are relatively exclusive to the fermentative bioprocessing bacteria that possess few hemicellulases (Sastry et al. 2019; Lim et al. 2022). However, this approach has yielded interesting results for Gram-positive species. For example in *C. thermocellum*, a LacI transcriptional regulator (GlyR2) was computationally predicted as important for genes encoding two mannanases (*man5A* and *man26A*), a xylanase (*clo1313_2530*), and two cellulases (*clo1313_0413* and *clo1313_1425*) (Wilson et al. 2017; Hebdon et al. 2021). Previous experimental research on GlyR2 had identified it as a mannobiose-responsive transcriptional repressor with only confirmed regulatory activity on a mannanase-encoding gene (*man5A*) (Wilson et al. 2017). GlyR2 was hypothesized to have indirect effects on the transcriptional regulation of certain hemicellulose-encoding genes that may require different conditions to de-repress other genes with the recognized binding motif (Hebdon et al. 2021). Additionally, a *C. bescii* genome analysis and comparison to other *Caldicellulosiruptor* species improved the organism-specific bioprocessing model through the discovery of 16 key regulators important to the degradation and metabolism of hemicellulose and pectin (Rodionov et al. 2021). It was noted that most of these regulators were involved in the expression of xylanase or pectinase-encoding genes, while genes that encoded cellulases, mannanases, and amylases generally only had one regulator for each CAZyme type. Additionally, the mechanistic regulatory actions of the predicted regulators were overwhelmingly repressive in function with the few activators belonging to the AraC family. Interestingly, the study found that most of these activators were involved in the regulation of pectinase-encoding genes.

## Future directions

A thorough understanding of how hemicellulase-encoding genes are regulated is essential to optimize lignocellulose bioprocessing (Chettri et al. 2020). Consequently, detailed studies that include hemicellulase-encoding gene regulation are generally conducted exclusively on well-characterized model bacteria and those already being used as chassis in biotechnology applications (Xiong et al. 2018; Rodionov et al. 2021).

Given that metagenomic and metatranscriptomic data for less characterized lignocellulolytic bacteria with unoptimized systems are available (Dai et al. 2015; Kougias et al. 2018; Lopez-Mondejar et al. 2020) but beyond the scope of this review, we have endeavored to summarize and highlight the current state of hemicellulose-encoding gene regulation patterns between Gram-positive and Gram-negative bacteria.

Overall, we argue there are two critical features of hemicellulase-encoding gene regulation that must be considered for optimization, which are (1) identifying the specific metabolic inducer (often an oligosaccharide), and (2) mitigating the impacts of carbon catabolite repression. Current lignocellulose bioconversion systems typically use Gram-positive species for saccharification and Gram-negative species for fermentation (Dai et al. 2015; Thapa et al. 2019). While it has been previously argued that co-culture or consortia-based bioconversion processes will improve the efficiency and completeness of lignocellulose degradation (Chin et al. 2020; Kumar et al. 2023), the amount of strain engineering and optimization significantly increases for each strain added to the process, especially given the current trend of focusing only on improving either degradative or metabolic/fermentative capabilities. Therefore, the following commentary will focus exclusively on the optimization of single bacterium bioprocessing systems for the complete deconstruction and utilization of lignocellulose (Table 1).

**Table 1:** Current limitations of select bacterial bioprocessors and suggested research approaches

	Bacterium	Current limitations	Suggested approach
Gram-positive	*Clostridium thermocellum*	• Requires synthetic biology to utilize non-cellulose-derived sugars	Improve genetic tools to control regulation of heterologously expressed genes
		• Engineered fermentation pathways for plant sugars repressed by plant oligosaccharides	Improve transcriptional control over heterologously expressed genes
	*Caldicellulosiruptor bescii*	• Low expression and degradative efficiency of heterologously expressed CAZyme-encoding genes	
Gram-negative	*Cellvibrio japonicus*	• No high-yielding, stable plasmid system for gene expression	Develop a stably replicating plasmid for gene expression
		• Does not produce any current high-value metabolite in abundance	
	*Saccharophagus degradans*	• Poor genetic system	Develop genetic tools to engineer a commodity product-producing strain
		• Cannot natively ferment sugars to fuels and/or renewable chemicals	

### Optimizing Gram-positive systems will require integration of degradative and fermentative capabilities

*Clostridia* and *Caldicellulosiruptor* species are highly studied genera for their prolific degradation of plant polymers (Artzi et al. 2018; Brunecky et al. 2018; Williams-Rhaesa et al. 2018). However, neither system has been successfully engineered to fully metabolize and ferment all components of lignocellulose. In the case of *Clostridia* systems, this is due to an inherent inability to ferment pentoses. A previous attempt to engineer a pathway for xylose fermentation in *C. thermocellum* found that while xylose and Avicel could be co-utilized, xylan and Avicel could not (Xiong et al. 2018). It was argued that this is likely due to an inhibitory effect posed by cello-oligosaccharides on xylanases or unknown regulators that repress xylanase gene expression in the presence of cello-oligosaccharides. More recently, efforts have transitioned to develop CRISPR/Cas systems or riboswitches (Marcano-Velazquez et al. 2019; Walker et al. 2020) to mediate the observed repression of xylanase gene transcription in the presence of cellodextrins or cellobiose*.*

In *Caldicellulosiruptor* systems, the limiting factor is that the expression and degradative efficiency of heterologously expressed CAZymes is low. *C. bescii* has been extensively manipulated to improve its saccharifying proficiency via heterologous expression of xylanases (Kim et al. 2018; Crosby et al. 2022); however, it has been observed that degraded oligosaccharides repress the expression of secreted enzymes. Additionally, many heterologously expressed genes in *C. bescii* employ a highly active constitutive promoter, which is unoptimized for lignocellulose bioprocessing due to the energetic output required to constitutively and highly express heterologous CAZyme-encoding genes (Conway et al. 2017; Kim et al. 2017; Lee et al. 2020). Therefore, control over the expression of the heterologously expressed genes could spare the metabolic burden of their high expression levels and improve this limitation.

### Optimizing Gram-negative systems will require bolstering the potency of lignocellulolytic capabilities

Gram-negative species elicit a much broader hemicellulase-encoding gene regulatory response than Gram-positive bacteria. We argue that this diversification of CAZyme gene expression is an underutilized resource to optimize lignocellulose bioconversion in single bacterium systems. Biotechnology-relevant model systems like *E. coli* and *P. putida* have been largely focused on improving co-utilization of hexoses and pentoses by overcoming the effects of CCR (Kim et al. 2019b; Peabody et al. 2019; Elmore et al. 2020; Cabulong et al. 2021). However, these systems are limited as they are unable to innately degrade lignocellulose. The necessary step needed to drive either model into a fully self-sufficient system is the inclusion of lignocellulolytic machinery. This approach has several obstacles, most pressingly, identifying the minimally sufficient set of CAZymes that can completely depolymerize plant biomass and engineering an efficient export system for these CAZymes from the heterologous host.

In contrast, the genes/proteins needed to ferment plant sugars or produce other bioproducts are known and could be integrated into lignocellulolytic Gram-negative species. One example of a system not yet tapped for industrial use but has to potential to do so is *Cellvibrio japonicus*, a Gram-negative saprophyte that can fully degrade lignocellulose (Deboy et al. 2008; Gardner et al. 2014; Larsbrink et al. 2014; Blake et al. 2018). *C. japonicus* has also been shown to make ethanol and rhamnolipids as targeted products from lignocellulose bioconversion on a proof-of-concept scale (Gardner and Keating 2010; Horlamus et al. 2018). Another Gram-negative model is *Saccharophagus degradans* which also possesses a large number of CAZymes capable of degrading polysaccharides including cellulose, xylan, and pectin (Ensor et al. 1999). Engineering efforts using *S. degradans* have successfully generated strains capable of producing polyhydroxyalkanoate (PHAs) from cellulose, xylan, and agarose (Esteban Alva Munoz and Riley 2008; Sawant et al. 2017). However, *S. degradans* cannot generate ethanol and still relies on co-culture with other microbes for its production (Takagi et al. 2016). While both *C. japonicus* and *S. degradans* show promise with their degradative ability, improvements to their genetic systems are still needed to heterologously express the necessary metabolic pathways to produce high-value products.

## Concluding statement

This review discussed mechanisms that regulate hemicellulase-encoding gene expression in Gram-positive versus Gram-negative bacteria. Experimental studies that characterize the molecular mechanisms of hemicellulase gene expression are useful to identify relevant activators or repressors for each regulon, and we argue that such research is essential for the field to significantly advance. Given the discussed limitations of the reviewed models, the field should prioritize efforts that predict transcriptional regulatory networks and engineer the requisite enzymes for plant sugar bioconversion in species innately capable of prolific lignocellulose degradation.

## Data Availability

N/A.
